# Quantification of the Psychoacoustic Effect of Noise from Small Unmanned Aerial Vehicles

**DOI:** 10.3390/ijerph18178893

**Published:** 2021-08-24

**Authors:** C. T. Justine Hui, Michael J. Kingan, Yusuke Hioka, Gian Schmid, George Dodd, Kim N. Dirks, Shaun Edlin, Sean Mascarenhas, Young-Min Shim

**Affiliations:** 1 Acoustics Research Centre, Department of Mechanical Engineering, The University of Auckland, Auckland 1010, New Zealand; justine.hui@auckland.ac.nz (C.T.J.H.); y.hioka@auckland.ac.nz (Y.H.); g.schmid@auckland.ac.nz (G.S.); g.dodd@auckland.ac.nz (G.D.); 2 Department of Civil and Environmental Engineering, The University of Auckland, Auckland 1010, New Zealand; k.dirks@auckland.ac.nz; 3 Dotterel Technologies Ltd., 40 Kenwyn Street, Parnell, Auckland 1052, New Zealand; shaun.edlin@dotterel.co.nz (S.E.); sean.mascarenhas@dotterel.co.nz (S.M.); young-min.shim@dotterel.co.nz (Y.-M.S.)

**Keywords:** unmanned aerial vehicles, vehicle noise, psychoacoustic annoyance

## Abstract

This paper presents the results of a study evaluating the human perception of the noise produced by four different small quadcopter unmanned aerial vehicles (UAVs). This study utilised measurements and recordings of the noise produced by the quadcopter UAVs in hover and in constant-speed flight at a fixed altitude. Measurements made using a ½″ microphone were used to calculate a range of different noise metrics for each noise event. Noise recordings were also made using a spherical microphone array (an Eigenmike system). The recordings were reproduced using a 3D sound reproduction system installed in a large anechoic chamber located at The University of Auckland. Thirty-seven participants were subjected to the recordings and asked to rate their levels of annoyance in response to the noise, and asked to perform a simple cognitive task in order to assess the level of distraction caused by the noise. This study discusses the noise levels measured during the test and how the various noise metrics relate to the annoyance ratings. It was found that annoyance strongly correlates with the sound pressure level and loudness metrics, and that there is a very strong correlation between the annoyance caused by a UAV in hover and in flyby at the same height. While some significant differences between the distraction caused by the UAV noise for different cases were observed in the cognitive distraction test, the results were inconclusive. This was likely due to a ceiling effect observed in the participants’ test scores.

## 1. Introduction

Unmanned aerial vehicles (UAVs) are becoming increasingly prevalent in communities around the world. Current and future applications of UAVs include aerial photography and filming, search and rescue, hobby flying and parcel delivery. Torija and Clark [1] cite several studies [2,3,4] that claim that the use of battery-operated UAVs for cargo transport and parcel delivery is expected to significantly increase in the future and that this will significantly reduce greenhouse gas emissions associated with these activities. However, they note that there are a number of issues which need to be addressed in order to allow this to occur. These include safety concerns, airspace management considerations, visual impact and noise impact.

The noise generated by UAVs is unique and does not resemble the noise produced by other common community-noise sources such as automobiles and conventional aircraft [5,6]. Because of their likely use in highly populated areas, UAV operations can be expected to alter existing soundscapes and thus affect people living within these areas [6]. Torija and Clark [1] state that “noise is one of the main limiting factors for public acceptance and adoption of UAVs”. They also note that further research is needed to understand the effect of UAV noise on humans, and that there is a need to develop or identify metrics suitable for quantifying the impact of UAV noise on humans, define acceptable levels for UAV noise, and investigate the impact of noise on communities due to current and future UAV operations.

**Figure 1:**
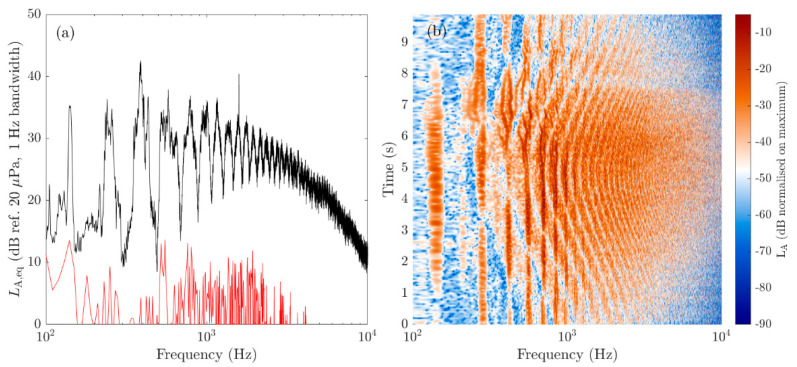
(**a**) *L*_A,eq_ spectrum produced by a hovering DJI Matrice UAV (black) with a representative background spectrum also shown (red); (**b**) A-weighted SPL spectrogram produced by a DJI Matrice in flyby. For both cases, the UAV was at a height 10 m above flat grass-covered ground and the noise was measured by a microphone 1.2 m above the ground and 10 m horizontally from the UAV position/flight path.

Figure 1a,b show the A-weighted narrow-band sound pressure level (SPL) spectrum and spectrogram of the noise produced by a DJI Matrice UAV carrying a 0.461 kg payload in hover and during flyby, respectively. The measurement method, microphone location and specifications of the UAV and its flight-path are described in detail in Section 2 and Section 3. The spectrum is observed to contain a significant number of sharp ‘peaks’ produced at harmonics of the blade passing frequency of the four rotors. The spectrogram shows that the frequency of these peaks can be slightly different (due to the rotors operating at slightly different speeds) and can vary throughout the flight. Additionally, clearly observed in the spectrogram is an interference pattern which we believe was caused by interference between the direct sound from the UAV and the sound reflected off the ground. Destructive interference associated with this effect causes significant reductions in sound pressure levels at certain frequencies and times and is likely also observed in the noise spectrum measured for the UAV in hover.

A significant number of papers have appeared in the recent literature investigating the noise produced by UAVs. Some report measurements of noise from UAVs in flight (e.g., [7,8,9,10,11]), from full or partial UAVs statically mounted in an anechoic chamber (e.g., [12,13,14]), and also from isolated UAV propellers statically mounted in an anechoic chamber with or without a flight stream (e.g., [15,16,17,18,19]). These and other investigations have identified that the noise produced by UAVs is mostly caused by the rotors and the electric motors which power them. The rotor noise is produced by physical sources including

- Steady loading and thickness noise sources. These generate tonal noise which is usually significant at the first two or three harmonics of the blade passing frequency and which radiates strongly close to the plane of the propeller [15,17];
- Periodic unsteady loading on the rotor blades produced by the aerodynamic effect of the adjacent rotors, the UAV body and the flight of the UAV can produce tonal noise [14,20];
- Turbulent inflow noise, which can produce significant ‘quasi-tonal noise’ at harmonics of the blade passing frequency. This noise source requires the flow incident on the propeller disc to be turbulent. Turbulent inflow noise can be predicted using a computational method such as that described in [21] or analytical methods such as that described by [22]. Note that tests conducted in anechoic chambers or confined spaces will produce a recirculating flow, which can produce high levels of turbulent inflow noise [23,24];
- The periodic unsteady blade motion caused by the pulsing motion of the propeller blades can also produce tonal noise [25];
- ‘Self-noise’ caused by the unsteady loading on the propeller blades generated by the turbulent flow in the boundary layers, near wake and tip vortex produced by the rotor blades can produce broadband noise at high frequencies [17];
- Blade vortex interaction and blade wake interaction noise caused by the rotor blades interacting with the tip vortex and wake from the preceding rotor blades can produce broadband noise [16].

In addition to noise from the rotors, the electric motors which power the rotors can also produce significant levels of noise at high frequencies. This typically contains a multitude of tones. Noise from these has been investigated in [26,27,28].

A number of studies have also investigated the psychoacoustic effects of noise from UAVs. For example, Christian and Cabell [5] describe a study in which a psychoacoustic test was undertaken in order to investigate the annoyance caused by UAV noise. This involved using a 3D sound reproduction system to expose 38 participants to sound recordings of four different multi-rotor UAVs performing straight-and-level flyovers at 5 m/s and 10 m/s at various altitudes above ground level. During these tests, the participants were asked to rate the annoyance of each sound recording. The correlations between the annoyance rating and a range of different sound metrics were then analysed. The analysis suggested that the A-weighted sound exposure level (*L*_EA_) and effective perceived noise level (EPNL) metrics correlated most closely with the subjective annoyance ratings—with the C-weighted sound exposure level (*L*_EC_) and 5th percentile loudness (*L*_5_) showing slightly lower correlation. It was also observed that the height of the UAV only weakly influenced the subjective annoyance rating and that this effect was not adequately reflected in the noise metrics (which suggested that annoyance should decrease significantly with increased UAV height). It was suggested that this effect could be partially due to the fact that noise events caused by UAVs at higher heights subjectively took longer to ‘die away’ than noise events produced by UAVs at lower heights. Subjects were also asked to rate the annoyance of road vehicles, which were generally rated as subjectively less annoying than the UAVs for recordings with comparable levels calculated using the various noise metrics. It was suggested that part of the reason for this could have been the shorter duration of the noise events in the road vehicle noise recordings. This argument is similar to a suggestion made by Senzig et al. [29] who state that because UAVs may hover for an extended period, the long duration of the noise from these operations may increase annoyance. However, Senzig et al. also state that, as small UAVs operate in close proximity to people, the “rise and fall times” of the noise they produce during a flyby event may be much shorter than with noise from conventional aircraft. Senzig et al. suggest that this may lead to noise from UAVs being perceived as more annoying due to a “startle effect”. Torija, Self and Lawrence [30] recorded the noise produced by a DJI phantom 3 quadcopter as well as several aircraft and road vehicles. Measurements of the UAV noise were taken both in an outdoor setting as well as in an anechoic chamber. All audio recordings were normalised to a common A-weighted equivalent continuous sound pressure level (*L*_A,eq_) to remove the effect of noise level on the analysis. For each recording, the following metrics were calculated: time-varying loudness (N), sharpness (S), roughness (R), fluctuation strength (F), tonality (T), and the psychoacoustic annoyance ratings (PA) of Zwicker and Fastl [31], Di et al. [32], and More [33]. The noise spectra produced by the quadcopter, aircraft and road vehicles were presented and the differences discussed. It was noted that the quadcopter produced a significant number of tones and that the aircraft noise spectrum contained little high frequency content relative to the quadcopter and road vehicle noise spectra due to atmospheric absorption of this sound. It was also found that the psychoacoustic annoyance ratings for the noise produced by the quadcopter were higher than those of both the road vehicles and aircraft. Torija et al. [6] reported on a study in which a series of audio-visual recordings were created to investigate the effects of UAV noise on the human perception of different urban soundscapes. These recordings were played to a number of persons using headphones and a virtual-reality headset in order to reproduce the listening environment accurately. Test participants were asked to report their perception of the soundscapes. It was found that in soundscapes with relatively low levels of traffic noise, participants reported being significantly annoyed by the UAV noise. However, UAV noise caused less annoyance in soundscapes with relatively high levels of road noise. Gwak, Han and Lee [34] reported on a psychoacoustic study investigating the subjective annoyance of noise produced by a number of different UAVs operating in hover. It was found that the noise produced by larger UAVs was generally perceived as most annoying. Participants in this study reported that in some of the noise recordings, the UAV noise could be described as “unpleasant”, “sharp” and “buzzing” and that these were related to the sharpness (S) and fluctuation strength (F) metrics. Torija and Li [35] presented the results of an experiment investigating the psychoacoustic effect of the noise produced by a quadcopter UAV during a straight-and-level flyover. It was found that both tonality and loudness-sharpness interaction were important for determining the preference rating of the quadcopter noise. Torija, Chaitanya and Li [36] present the results of a study in which the noise produced by a contra-rotating UAV propeller mounted statically in an anechoic chamber was measured. The noise produced by different propeller configurations was assessed using psychoacoustic metrics including loudness, fluctuation strength, roughness, sharpness, tonality, and several psychoacoustic annoyance models.

There has also been recent work investigating the suitability of current aircraft noise metrics to assess the impact and annoyance of non-standard aircraft noise. For example, Torija et al. [37] present the results of a listening test in which subjects were exposed to noise produced by different aircraft during take-off. It was found that the tonality noise metric was better than the traditional EPNL tone correction in assessing human preference for noise which contained multiple complex tones. These tones can be produced by conventional turbofan powered aircraft via the “buzz-saw” noise source [38]. Rizzi et al. [39] describe a study in which auralisations of the noise from a distributed electric propulsion aircraft was used in a psychoacoustic test in order to assess human annoyance caused by this sound. The annoyance caused by aircraft with different design features was assessed. A noise annoyance model was also developed which was dependent on the noise metrics of loudness, roughness and tonality. Torija and Clark [1] discuss the applicability of different noise metrics to assess the noise produced by UAVs. They state that traditional metrics such as the maximum A-weighted sound pressure level (*L*_A,max_), *L*_AE_ and EPNL are not ideal for assessing the human response to UAV noise as they do not adequately take into account the noise fluctuation strength nor the highly tonal nature of this noise. Read and Roof [40] also suggest that traditional metrics are not suitable because of the likely intermittent nature of UAV noise and also because of its frequency content.

Recently, there has been a significant effort to develop standard methods for assessing noise from UAVs. Senzig et al. [29] describe work performed to support the Federal Aviation Authority (FAA) of the USA to develop a noise certification and measurement method for unmanned aerial systems (UASs) (which include UAVs). This work involved developing an accurate system for tracking the position of the UAS during testing. Measurements of the noise produced during flyby of a large (180 kg) fixed-wing UAS were presented. These measurements were conducted in general accordance with the noise certification method described in [41]. They note that the noise produced by UASs is quite different to conventional aircraft and that conventional aircraft noise metrics may not correlate well with the actual annoyance that these aircraft cause. They recommend that further work is required to identify or develop suitable noise metrics which do correlate well with human annoyance to UAS noise. Senzig and Marsan [42] discuss the main limitations of applying noise certification methods such as [41,43] for conventional aircraft to UAS vehicles. They conclude that these “may not represent the best methods for certifying UAS vehicles in the future”. They also state that further investigation is needed into what flight operations best represent expected actual operations and would therefore be well suited for use in certification testing. Hellweg [44] discusses recent efforts in the USA to develop a noise certification method for small UAVs as part of an ANSI/ASA standard. Torija and Clark [1] provide a summary of the efforts to develop noise regulations for UAVs in Europe. They report that the European commission has issued a regulation [45] for assessing the noise produced by UAVs with masses less than 25 kg. This regulation requires that the A-weighted sound power level should be determined for a UAV hovering 0.5 m above an acoustically hard reflecting plane. The standard specifies maximum A-weighted sound power levels for UAVs based on their mass and their time in the market. Torija and Clark note that this test method does not take other flight operations into account, and that different operations (e.g., straight-and-level flight) will produce noise with different spectral and temporal characteristics. Weiland et al. [46] also discuss the development of this regulation and list issues and further work required. Recently, work has commenced on the development of an international (ISO) standard for specifying the noise measurement method of lightweight and small multi-rotor UASs. This work is being undertaken by a Joint Working Group comprising international experts drawn from ISO TC 20/SC 16 (Unmanned Aircraft Systems) and ISO TC 43/SC 1 (Noise) and is led by Prof Xin Zhang from the Hong Kong University of Science and Technology.

In this paper, we present the results of a study investigating the psychoacoustic effect of the noise produced by different small quadcopter UAVs. The overall purpose of this study was to investigate the annoyance of, and cognitive distraction caused by, UAV noise, and to identify metrics which are well suited to quantifying this noise. It is hoped that this work will be able to contribute to the development of a certification method for quantifying the noise impact of UAVs. This study involved measuring and recording the noise produced by several different UAVs operating in hover and straight-and-level flight at different heights. These noise recordings were played to human subjects using a higher-order Ambisonics (HOA)-based 3D sound reproduction system. Subjects were asked to rate the annoyance of the sounds they were listening to, and to also perform a cognitive task in order to assess whether the noise had any effect on cognitive function. For each noise recording, a number of different noise metrics were calculated. A statistical analysis of the annoyance rating, cognitive test performance and noise metrics for each noise recording/measurement was conducted in order to identify which metrics were most suitable for assessing the psychoacoustic effect of UAV noise.

Our research questions are as follows:

a. How annoying is the noise from different types of UAVs operating in different flight modes (i.e., hover vs. flyby, high vs. low altitude)?
b. What effect does UAV altitude have on the annoyance caused by UAV noise?
c. Is there a relationship between the annoyance caused by the noise produced by a hovering UAV and that produced by a UAV in flyby?
d. What metric correlates best with the annoyance caused by the noise from UAVs operating in either hover or in flyby?
e. How does drone noise affect performance during a cognitive distraction test?

The UAVs used in this study and the method used for recording, measuring and analysing the noise which they produce are described in Section 2 and Section 3. This is followed in Section 4 by a description of the sound reproduction system and the psychoacoustic test method used. The results of the psychoacoustic experiment are presented in Section 5 along with analysis and discussion. The conclusions are given in Section 6.

## 2. Quadcopter UAVs and Flight Conditions

**Figure 2:**
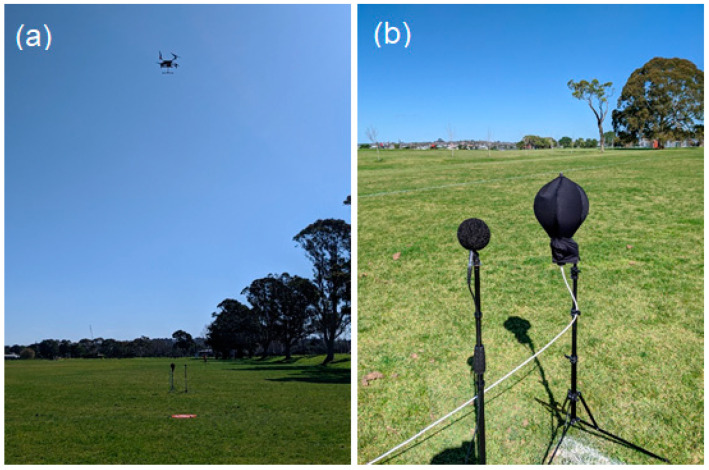
(**a**) The Matrice quadcopter UAV hovering above the two microphones; (**b**) the ½″ microphone and Eigenmike with wind socks mounted on tripods.

Four different commercially available DJI quadcopter UAVs were used in this study: a Mavic 2, a Matrice 210 V2, a Phantom 3, and a Tello. These 4 UAVs have nominal weights of 0.907, 4.8, 1.216, and 0.08 kg, respectively. All UAVs were tested in their standard configuration. The Mavic was also tested with an alternative set of ‘low-noise’ rotors, and the Matrice was also tested with a 0.461 kg payload. All UAVs were tested operating in hover, and all but the Phantom were tested performing a straight-and-level flight travelling at approximately 5 m/s. These tests were run at heights above ground level of 10 and 30 m. Because of difficulties controlling the Phantom, the Phantom was tested at a height of 27 m instead of 30 m. Additionally, the Tello was only tested at a height of 10 m. The height of the UAV and the flight speed were set using the control system supplied with the UAV, typically consisting of lidar and optical flow sensors, a barometer, and GPS to determine the UAV’s height and flight speed. This information was displayed for the pilot. The pilot maintained the flight speed by holding a constant throttle level once the craft reached 5 m/s. A photograph of a UAV in hover during testing is shown in Figure 2a. Table 1 contains the details of the tests which were performed.

## 3. Noise Recording/Measurement and Analysis

For the purpose of accurately measuring the noise levels produced by the UAV in flight, measurements were made using a GRAS 46AE ½″ free-field microphone with preamplifier connected to a Zoom H6 recorder recording at 48 kHz with 24-bit resolution. The microphone was mounted on a tripod with its diaphragm 1.2 m above ground level, with a windsock used to minimise wind noise during measurements. The microphone was calibrated prior to the UAV noise recording/measurement tests. The noise from the UAVs was also recorded using an MH Acoustics Eigenmike which was covered in a windsock supplied by the manufacturer and located adjacent to the ½″ microphone. The Eigenmike is a spherical microphone array which was used in this study to record the 3D sound field at the Eigenmike’s location. These recordings were used to generate an accurate reproduction of this sound field using the 3D sound reproduction system employed during the psychoacoustic tests. The Eigenmike records 32 channels of audio data at a frequency of 44.1 kHz and with 24-bit resolution. Photographs of the microphones during the recording/measurement tests are shown in Figure 2b.

The noise recordings/measurements were all made on the 8 September 2020 at Point England Reserve, an urban park located in the city of Auckland, New Zealand. The UAV noise measurement/recording test location was well away from any road traffic. Although noise from birds and people using the park could occasionally be heard. Measurements/recordings were made during times when noise from such sources was minimal. Moreover, the background noise was much lower in level than the noise produced by the UAVs during measurement/recording. A representative background noise level is shown in Figure 1a. The weather during the measurement/recording tests was fine and with a light North/Northeasterly wind.

For all measurement/recording tests, the UAVs hovered or flew along a flightpath situated approximately 10 m horizontally from the location of the microphone. A white line painted on a sports field at the park was used to help the pilot maintain the UAV flying in a straight line during the straight-and-level measurement/recording tests.

The data from the calibrated ½″ microphone were processed and used to calculate the various noise metrics for each of the measurement/recording tests. For hover measurements, the following noise metrics were calculated:
*F*_5_5th percentile of the time-varying fluctuation strength in vacil calculated according to the methods described in [31] and ISO 532-1 [47] and calculated using Matlab’s Audio Toolbox; *L*_A,eq_A-weighted continuous equivalent sound pressure level;*L*_A,f,max_A-weighted maximum sound pressure level (using a fast time weighting);*L*_A,f,5_A-weighted sound pressure level exceeded 5% of the time (using a fast time weighting);*L*_PN,m_Mean perceived noise level in dB calculated using the method described in Part 36 of the noise certification requirements of the United States Code of Federal Regulations; *L*_PN,5_5th percentile perceived noise level in dB calculated using the method described in Part 36 of the noise certification requirements of the United States Code of Federal Regulations;*L*_TPN,m_Mean tone-corrected perceived noise level in dB calculated using the method described in Part 36 of the noise certification requirements of the United States Code of Federal Regulations;*L*_TPN,5_5th percentile tone-corrected perceived noise level in dB calculated using the method described in Part 36 of the noise certification requirements of the United States Code of Federal Regulations;*N*_m_Mean time-varying loudness in sones based on Zwicker’s method and described in ISO 532-1 [47] and calculated using Matlab’s Audio Toolbox; *N*_5_5th percentile of the time-varying loudness in sones based on Zwicker’s method and described in ISO 532-1 [47] and calculated using Matlab’s Audio Toolbox; PAZwicker psychoacoustic annoyance (see, for example, [36]) calculated as $\mathrm{PA}=N_{5}\left(1+\sqrt{w_{S}^{2}+w_{FR}^{2}}\right)$, where $w_{S}=0.25\cdot (S_{5}-1.75)\log_{10}(N_{5}+10)$ if $S_{5}>1.75$ and $w_{S}=0$ if $S_{5}\leq 1.75$; and $w_{FR}=2.18\cdot {\left(N_{5}\right)}^{-0.4}\cdot \left(0.4F_{5}+0.6R_{5}\right)$;*R*_m_Mean time-varying roughness in aspers based on the method described by [31] and ISO 532-1 [47] and calculated using Matlab’s Audio Toolbox; *R*_5_5th percentile of the time-varying roughness in aspers based on the method described by [31] and ISO 532-1 [47] and calculated using Matlab’s Audio Toolbox; *S*_5_5th percentile of the time-varying sharpness in acums calculated according to DIN 45692 [48] and ISO 532-1 [47] and calculated using Matlab’s Audio Toolbox.

For flyby, the *F*_5_, *L*_A,f,max_, *N*_5_, PA, *R*_5_, and *S*_5_ metrics were calculated along with:
*L*_EA_A-weighted sound exposure level calculated over the period in which the noise level was within 10 dB of the maximum A-weighted sound pressure level, or over the entire recording period if the level never dropped below 10 dB of the maximum A-weighted sound pressure level; *L*_TPN,max_Maximum tone-corrected perceived noise level in dB calculated using the method described in Part 36 of the noise certification requirements of the United States Code of Federal Regulations.

## 4. Psychoacoustic Test Procedure and Data Analysis

This section describes the psychoacoustic experiment used in this study to determine the subjective annoyance rating of, and cognitive distraction caused by, each of the noise recordings. The statistical methods used to analyse the data collected during these experiments are also described.

### 4.1. Participant Group

Thirty-seven people participated in the experiment. Participants were sought by advertising at the University of Auckland and also through emailing members of the Acoustical Society of New Zealand. Thus, the vast majority of participants were either students or staff at the University of Auckland or were members of the Acoustical Society of New Zealand (who were primarily practicing acoustical engineers). This group had a mean age of 29.8 years with a standard deviation of 10.8 years. Of these, nine identified as female, 28 as male and none identified as gender diverse. This study was restricted to participants who self-reported having no diagnosed hearing impairment. All were offered a small monetary *koha* (a gift) in return for their participation. The experiment was approved by the University of Auckland’s Human Participants Ethics Committee (Application Number: UAHPEC3215).

### 4.2. Sound Reproduction

The psychoacoustic experiment involved playing the UAV noise recordings to each participant individually. For this purpose, the noise recordings made using the Eigenmike were processed so that they could be rendered by a 16-channel spherical loudspeaker array to produce a 3D sound field, closely recreating the actual sound field at the location of the Eigenmike during recording.

The A-format noise recordings made using the Eigenmike were encoded and decoded using the digital audio software package, Cockos Reaper with the Spatial Audio Real-Time Applications (SPARTA) virtual studio technology (VST) plug-in suite [49]. The recordings were first encoded into third-order spherical harmonics using the *array2sh* plug-in [50] and then decoded using the *ambiDEC* plug-in configured to the layout of the loudspeaker array used in the experiment. The files created using this process were played through the loudspeaker array using a MOTU 16A audio interface.

**Figure 3:**
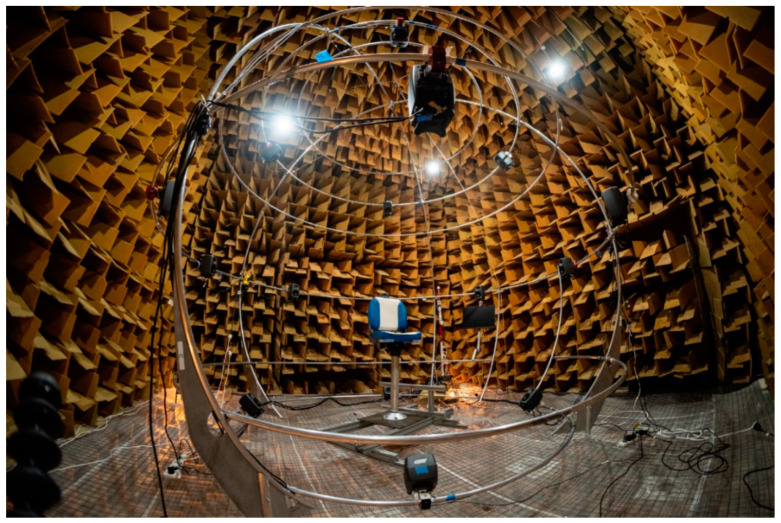
Sixteen-channel spherical loudspeaker array setup in the anechoic chamber at the University of Auckland (taken from [51]).

**Figure 4:**
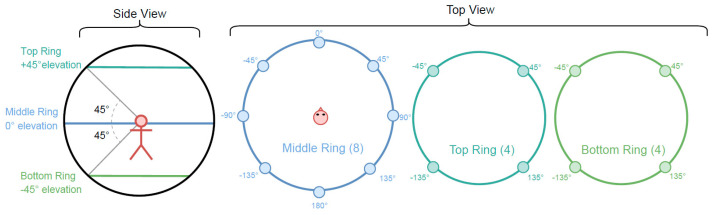
Side and plan view schematics of the loudspeaker array layout. Shaded circles denote the loudspeaker locations and the labels indicate the azimuthal angle of the speaker (taken from [51]).

The loudspeaker array was mounted on a spherical wire-frame structure, which was custom-built from 25 mm diameter aluminium tubing and installed within the anechoic chamber at the University of Auckland. This chamber has large acoustic wedges on all interior surfaces and has a cut-off frequency of approximately 80 Hz and a negligible reverberation time. During the experiment, participants sat on a chair, with the height of the chair adjusted so that their head was located at the geometric centre of the loudspeaker array. A photograph of the loudspeaker array and chair installed in the anechoic chamber is shown in Figure 3. Figure 4 shows a schematic of the loudspeaker array layout and indicates the azimuthal angles at which the loudspeakers were located. The wireframe support structure consists of three horizontal rings onto which the loudspeakers were attached. Four loudspeakers were attached to the top ring, eight to the middle ring, and four to the bottom ring.

The level of the noise produced by the array was adjusted so that, for each noise recording, the maximum A-weighted sound pressure level measured at the centre of the loudspeaker array corresponded with that measured by the ½″ microphone during the UAV noise measurement/recording tests. The background noise level within the anechoic chamber during testing was very low (we believe with an *L*_Aeq_ < 10 dBA). Note that ethical approval was granted for this study on the basis that the chamber door would be left slightly ajar during testing.

### 4.3. Administration of the Questionnaire

A questionnaire was used to collect noise annoyance ratings from the test participants and to administer a cognitive distraction test. This questionnaire was administered via a graphical user interface (GUI) displayed on a computer monitor placed directly in front of the participant. The GUI was developed in Python and interfaced with the Cockos Reaper audio software, controlling the playback of the UAV noise recordings and saving the participants’ responses. Note that the computer monitor is not shown in either Figure 3 or Figure 4, but was installed just below the loudspeaker located at an azimuthal angle of 0° (see Figure 4). Participants entered their answers to the questionnaire using a wireless keyboard.

Upon arrival, participants were asked to fill in a pre-experiment questionnaire and were then taken into the anechoic chamber and seated in a chair located in the middle of the loudspeaker array. The height of the chair was adjusted to ensure that the participant’s ears were level with the cones of the loudspeakers attached to the middle ring and so that both ears were aligned with the loudspeakers located at azimuthal angles of ±90° (see Figure 4).

All participants completed the annoyance rating test followed by the cognitive distraction test. In order to ensure that the test participants were comfortable with the test procedures, a practice trial of both tests was carried out while the experimenter was in the room. Participants proceeded to the actual tests once they confirmed that they were confident with the test procedures.

For the annoyance rating test, participants were played a 10 s UAV noise recording and were asked to answer the question: “Imagine you are exposed to this noise from a flying UAV whilst outside in a public green space which is otherwise peaceful. How much would this noise bother, disturb or annoy you?”. They answered the question using a numeric slider on the GUI which had values ranging from 0 (“Not at all”) to 10 (“Extremely”), in increments of one. The test administered was based on the recommendations according to the standardised noise reaction questions in [52]. The question incorporated the importance of location, as recommended in [52], whilst keeping to a one-question format and numeric rating response, consistent with the approach described in [5]. The participant rated the annoyance after listening to the entire recording. All recordings were trimmed so that their duration was 10 s.

After the annoyance rating test, the participants proceeded to the cognitive distraction test. This took the form of a ‘digit-span test’ [53] to assess how each noise recording affected the working memory of the participant. In this test, participants were asked to memorise a randomly-generated nine-digit number displayed visually on the GUI, whilst one of the UAV recordings was presented aurally. After the 10 s noise recording had finished, there was a 5 s ‘retention period’ before participants were able to enter their response. This test was similar to that used in [54].

For both tests, each of the 20 noise recordings listed in Table 1 were played to the participant a total of three times. The cognitive distraction test also included two extra conditions: ‘silence’ and ‘ambient noise’. In these cases, instead of the UAV recordings being played, the participant was either exposed to silence or ambient noise recorded at the location where the UAV noise recordings were made. The silence and ambient noise conditions were added in order to obtain a baseline result from the participants for the cognitive distraction test. For each test, noise recordings were presented to the participant such that the hover noise recordings were all presented in one ‘block’ and the flyby recordings were presented in another block. The order in which either the hover or the flyby recordings blocks were played was counter-balanced between the participants. For example, if a participant heard the flyby recordings first in the annoyance test, then they would hear the hover recordings first for the cognitive distraction test. The order in which the noise recordings were played within each block of hover or flyby recordings was random for both the annoyance test and the cognitive distraction test.

In total, each of the participants was required to rate the annoyance of 60 separate UAV noise recordings (i.e., each of the 20 recordings specified in Table 1 repeated in three blocks randomly). In addition, for the cognitive distraction tests, they were required to carry out a total of 66 numeric memorisation trials (i.e., each of the recordings specified in Table 1 plus the ambient noise recording and the silence case repeated three times). The participants were given breaks during both the annoyance test and the cognitive distraction test, as well as between the two tests. Each participant took approximately 40 min to complete the experiment.

#### Statistical Analysis

The annoyance ratings and performance from the cognitive distraction test were analysed using a linear mixed effect (LME) model with the R package *lme4* [55,56] and model fitting was carried out using the step function from the R package *lmerTest* [55,57]. Interactions between two and more factors were included when it improved the fitness of the model. Post hoc pairwise comparisons of the models were carried out using the *emmeans* package [55,58] with *p*-values adjusted using the Tukey method. The *Hmisc* package [59] was used to calculate the Pearson’s product–moment correlation coefficients.

## 5. Results and Discussion

### 5.1. Noise Level Measurement Results

The noise metrics calculated from the ½″ microphone measurements showed that the level of the UAV noise strongly depended on the mass of the UAV, the distance from the microphone to the UAV, and the direction of the microphone relative to the UAV. Experimental investigations (e.g., [15]) have shown that the noise from a single UAV rotor is highly directional, with the sound pressure level being a function of the (polar) angle from the propeller axis and obeying the well-known spherical spreading law in the acoustic far-field. A quadcopter UAV operating in hover or flyby will have an even more complex directivity pattern, which will also likely vary with the (azimuthal) angle through which the propeller blades rotate due to the noise caused by the unsteady loading on the propeller blades and acoustic interference between the sound from each propeller. The levels measured during the experiments reported here are also affected by ground reflections (as shown in Figure 1).

**Figure 5:**
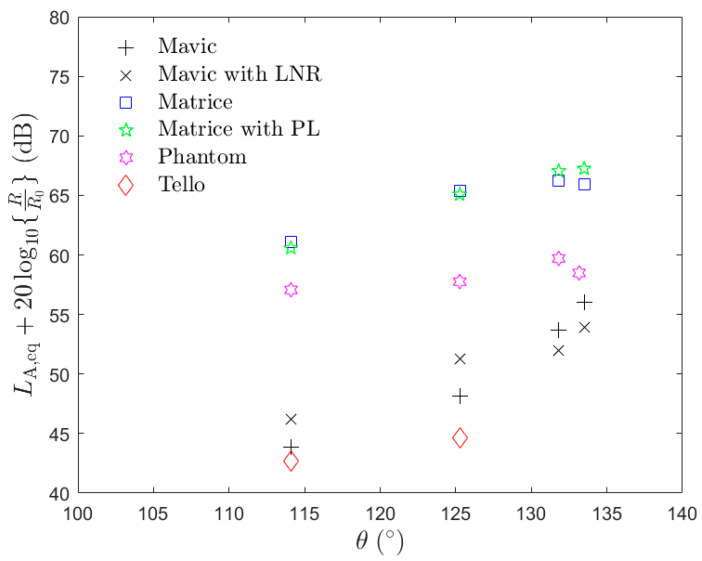
Plot of ‘distance-corrected’ *L*_A,eq_ versus polar angle θ, measured from the vertical.

Figure 5 plots the ‘distance-corrected’ *L*_A,eq_ calculated for each UAV operating in hover at different heights versus polar angle. The distance correction assumes that the noise from the UAV obeys the well-known spherical spreading law. Here, the polar angle is defined as the angle the ray from the microphone to the centre of the UAV makes with the vertical axis—with θ= 0° being directly above the UAV, θ= 90° being in the plane of the UAV rotors and θ= 180° being directly below the UAV. Note that additional data to those listed in Table 1 (with most of the UAVs operating at additional heights of 5 and 20 m) are included in this plot. The plot shows that the noise levels produced by all UAVs, except the Phantom, are generally higher further away from the plane of rotation of the propellers (θ= 90°) than would be expected assuming spherical spreading. This could be caused by the directivity of the sound from the UAV, but ground reflections may also have contributed to this effect.

**Figure 6:**
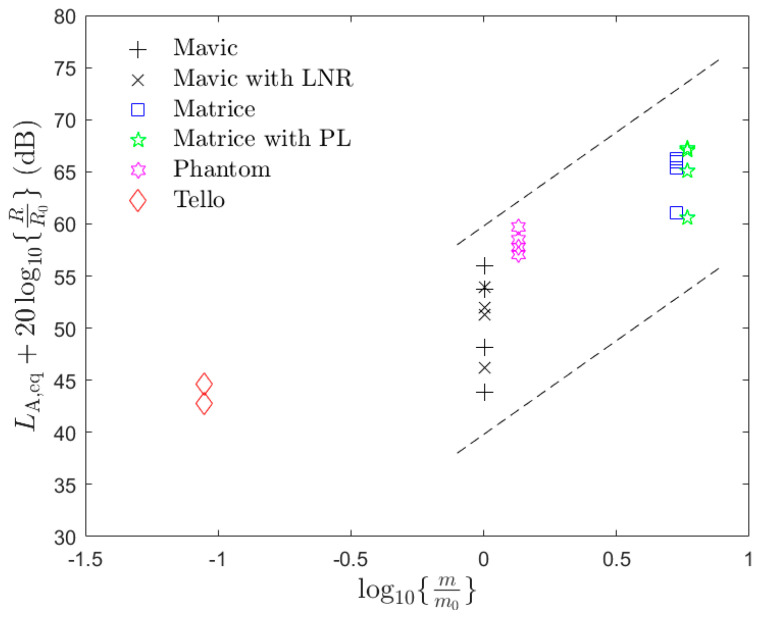
Plot of ‘distance-corrected’ *L*_A,eq_ versus $\log_{10}\left(m/m_{0}\right)$. m is the nominal mass of the UAV, $m_{0}=0.9$ kg is a reference mass, R is the distance between the UAV and the microphone location and $R_{0}=10\sqrt{2}$ m is a reference distance. The straight lines have gradients of 1.8, consistent with the scaling law in [45].

Figure 6 plots the distance-corrected *L*_A,eq_ calculated for the UAVs operating in hover at different heights versus $\log_{10}\left(m/m_{0}\right)$, where m is the nominal mass of the UAV, and $m_{0}$ is a reference mass of 0.9 kg. Straight dashed lines defined by $L_{A,\mathrm{eq}}\propto \log_{10}\left(m/m_{0}\right)$ are also plotted. This scaling is identical to that used in the EU regulation for noise from UAVs [45] for the maximum A-weighted sound power level produced by a UAV, hovering 1 m above the ground, for 0.9 kg≤m<4 kg. The data appear to be consistent with this scaling law, with the noise level increasing as UAV mass increases.

### 5.2. Annoyance Rating Analysis

**Figure 7:**
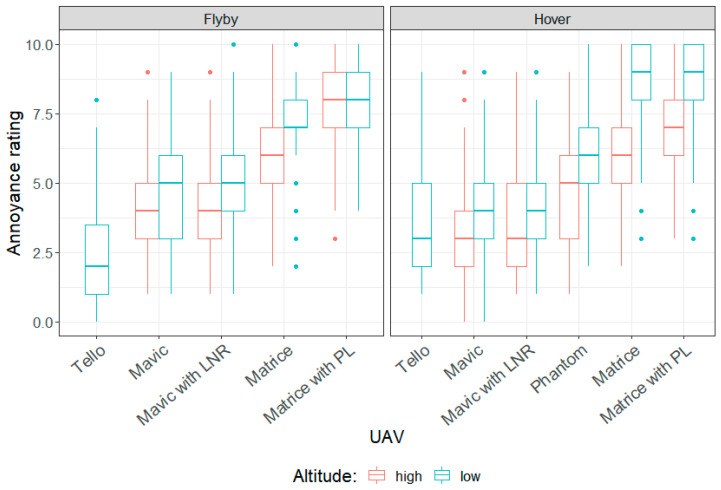
Responses from annoyance rating test.

Figure 7 presents box plots of the results of the annoyance rating test. Annoyance ratings for the different types of UAV flown in both hover or flyby modes, and at high altitude (30 m or 27 m) or low altitude (10 m), are presented. The reader will recall that no data were collected for the Tello operating at high altitude, nor the DJI Phantom operating in flyby mode and that a high annoyance rating corresponded to a participant perceiving a UAV noise event to be more annoying than an event with a lower rating.

A model with a three-way interaction between the UAV type, altitude, and flying mode (hover or flyby) as fixed effects was used to analyse the annoyance rating data. The participant ID was included as a random effect to account for the repeated measure in the test design. No random slope was included as it caused a singular fit of the model. We found a significant three-way interaction using the model (χ^2^(3) = 39.3, *p*-value < 0.0001) from a likelihood ratio comparison by comparing the full model with a model without the interaction.

Table A1 and Table A2 present the respective pairwise contrasts of the three-way interaction using *emmeans* within and between the UAV types.

**Figure 8:**
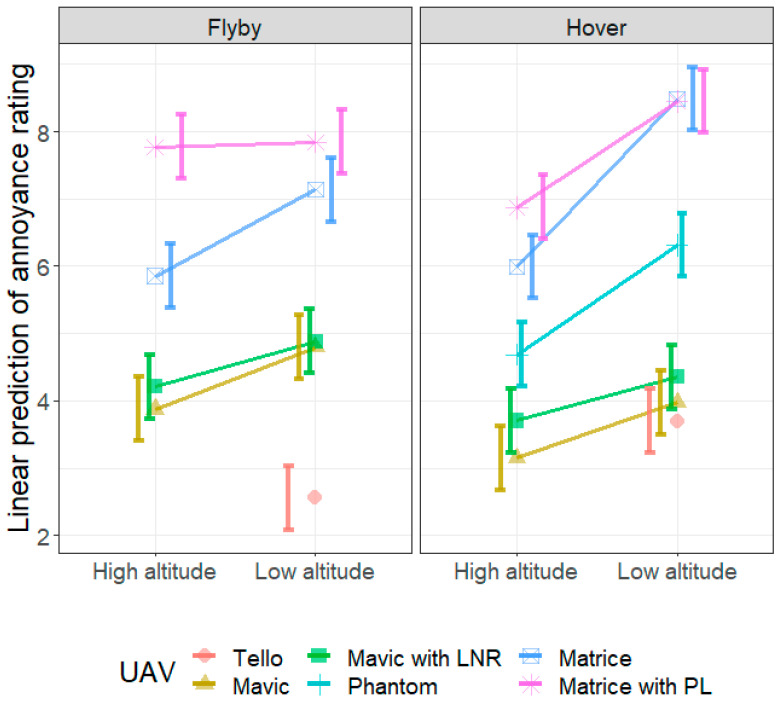
Linear prediction of the annoyance rating from the linear mixed model analysis. The effect of altitude for each UAV in flyby (**left**) and hover (**right**). The error bars represent the 95% confidence interval of the linear prediction.

Figure 8 shows the annoyance rating predicted using the LME model, with the data presented to show the predicted effect of altitude on the annoyance rating of the sound produced by each UAV. For all cases, the model predicts that the noise produced when the UAV is at low altitude will be more annoying than the corresponding case (same UAV and flight condition) at high altitude. However, this effect does not seem to be significant for the Matrice with payload during flyby. This is confirmed by the post hoc comparison in Table A1, where apart from the Matrice with PL in flyby, all contrasts between the predicted annoyance ratings for a particular UAV at high and low altitudes for a single flying condition were significantly different, with the low-altitude cases having a significantly higher annoyance rating.

**Figure 9:**
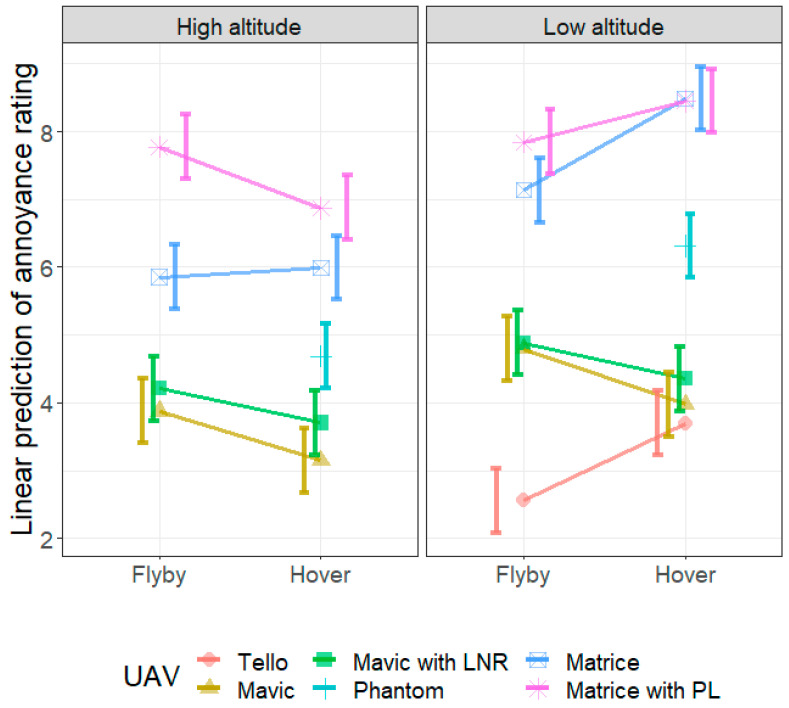
Linear prediction of the annoyance rating from the linear mixed model analysis. The effect of flying condition for each UAV in high (**left**) and low (**right**) altitudes. The error bars represent the 95% confidence interval of the linear prediction.

Figure 9 shows the annoyance rating predicted using the LME model, with the data presented to show the effect of the flying mode (hover or flyby) on the annoyance rating. There are significant differences between the annoyance caused by hover and that caused by flyby for all UAV types at both altitudes—apart from that caused by the Matrice operating at high altitude. At high and low altitudes, participants rated the Mavic as more annoying during flyby compared to hover. Similar to the Mavic, participants found the Mavic with LNR at both high and low altitudes, and the Matrice with PL at high altitude, to be more annoying during flyby compared to hover. However, the opposite was observed for the Matrice with and without PL, and the Tello, at low altitude—where flyby was less annoying than hover. These results suggest that, whether hovering is more or less annoying than flyby depends on the altitude and UAV type., i.e., for some cases (a particular UAV operating at a high or low altitude) hovering will be more annoying than flyby, and for other cases the opposite will be true.

The differences in annoyance ratings between the various UAVs are given in Table A2. As expected, the different UAVs produce noise which causes different levels of annoyance for identical flight mode and altitude. Generally, the noise produced by the larger UAVs causes more annoyance. There was also only a slight difference between the annoyance caused by the Mavic with and without the LNRs, with the Mavic with LNRs surprisingly being rated as slightly more annoying. Note that the *L*_A,f,max_ measured for the Mavic with LNRs was within 4 dB of the level measured for the Mavic without LNRs for all corresponding cases. There was also no evidence of significant differences between the annoyance produced by the noise from the Mavic (*L*_A,f,max_ = 51.8 dB) and the Tello (*L*_A,f,max_ = 46.1 dB), and between the Matrice with payload (*L*_A,f,max_ = 66.8 dB) and without payload (*L*_A,f,max_ = 66.8 dB) when hovering at low altitude. The fact that the annoyance produced by noise from the Matrice with and without payload was similar is consistent with the fact that the *L*_A,f,max_ values are similar. However, it is interesting that the annoyance produced by the Mavic and Tello are similar given the large difference in the *L*_A,f,max_ values. This is presumably due to the different characteristics of the noise produced by these UAVs. All other contrasts were significant.

These results show that the annoyance caused by UAV noise depends on both the flying mode and the altitude of the UAV. There are a few generalisations we can make from the statistical analyses. Low-altitude cases were in general more annoying than high-altitude cases for the same UAV and flying condition. This is expected as the UAV is farther from the microphone for the high-altitude cases, and thus produces a lower sound level at the microphone location which is less annoying. An exception to this rule was the Matrice with PL operating in flyby mode for which there was no significant difference between the low- and high-altitude cases. The Matrice with PL in flyby at both high altitude and low altitude produced similar, and very high, annoyance ratings. This is consistent with the relatively high and similar noise levels measured for these cases (*L*_A,f,max_ = 67.2 and 70.6 dB, respectively). While the current study does not have an answer as to why that is, future work could include examining the characteristics of the signal, such as its temporal variation and spectral characteristics, and how flying conditions and altitude affect the UAV noise signal and how this influences annoyance. The Tello, being the smallest UAV in the current study, was deemed to be the least annoying. This is to be expected, as it also produced the lowest levels of noise. However, the annoyance produced by the Tello was not significantly different from the larger (and louder) Mavic when hovering at a low altitude.

**Figure 10:**
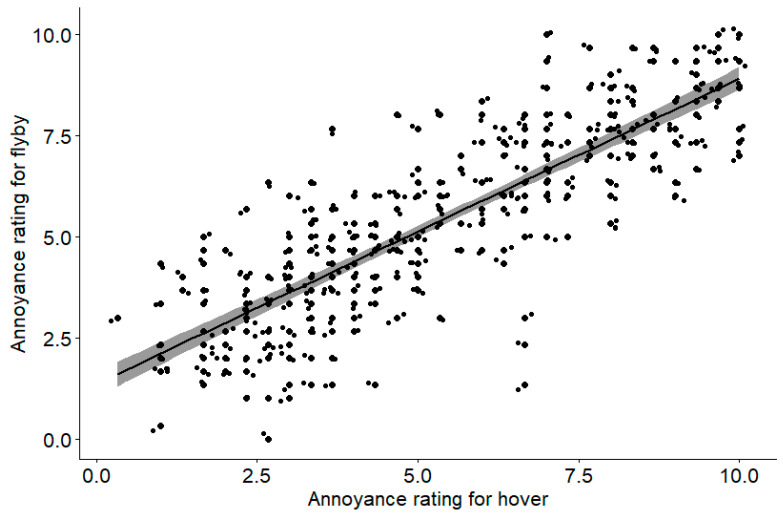
Correlation analysis between annoyance ratings in hover and flyby.

Figure 10 plots the mean annoyance ratings over three repetitions per participant for each flyby case against the annoyance rating for the corresponding hover case. Data for the DJI Phantom were omitted as there were no flyby data for this UAV. While there is a reasonable spread in the data, the results show that the annoyance ratings in hover are highly correlated with those in flyby (*r* = 0.83, *p* < 0.001). The linear fit in Figure 10 has an intercept of 1.36 and a slope of 0.75.

The strong correlation between the annoyance ratings for the noise produced by the UAV in hover and in flyby suggests that the noise produced by the different flying conditions may not be so important in determining how listeners perceive the annoyance of the noise produced by a UAV. Future work should examine how the flying condition affects the noise signal in terms of its spectral content and temporal variation, and how that may affect annoyance.

### 5.3. Correlation between Objective Measurements and Annoyance Ratings

**Figure 11:**
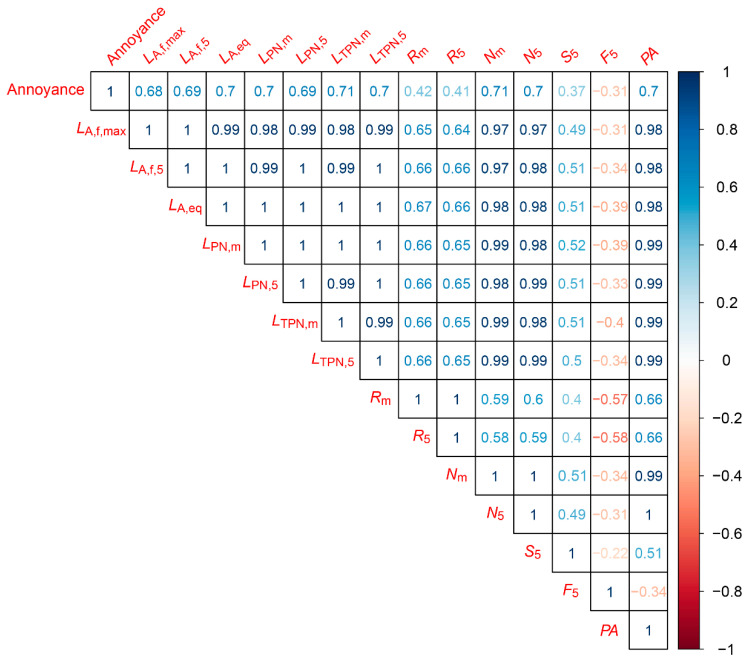
Correlation matrix between annoyance ratings and objective measurements for hover noise events.

**Figure 12:**
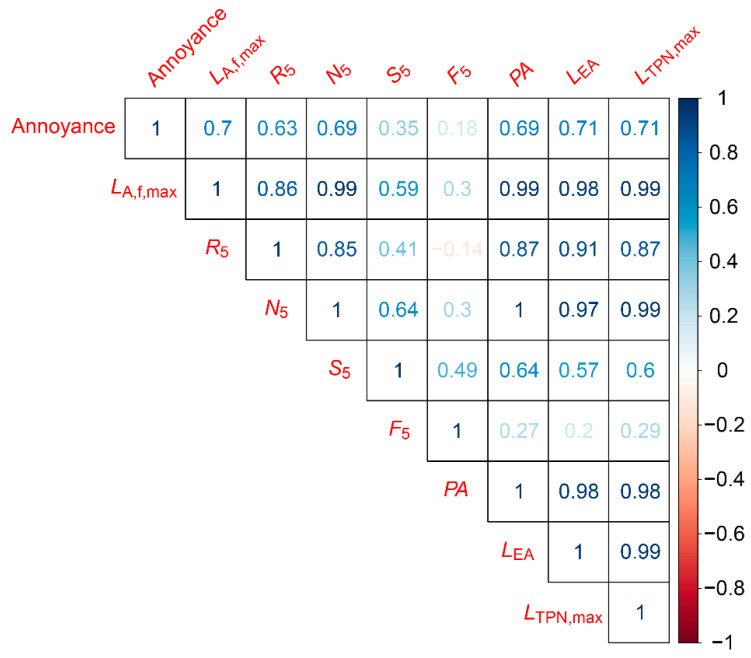
Correlation matrix between annoyance ratings and objective measurements for flyby noise events.

Figure 11 and Figure 12 present the correlations between the subjective annoyance ratings given by the participants and the various objective noise metrics calculated using the signal from the ½″ microphone. The numbers in the matrices are Pearson’s product–moment correlation coefficients (r), where the first row presents the correlation coefficients between the participants’ annoyance ratings and the metric. The remaining rows display the correlations between the different metrics. The opacity and colour of the numbers denote the strength (transparent or opaque) and the sign (blue or red) of the correlation.

Figure 11 presents the correlation coefficients between the annoyance ratings of the noise from the UAVs in hover and the various noise metrics. We see that the annoyance caused by the noise from a hovering UAV is strongly correlated with *L*_A,eq_, *N*_m_, *N*_5_, PA, *L*_PN,m_, *L*_TPN,m_, and *L*_TPN,5_ at r ≥ 0.7, followed closely by *L*_A,f,max_, *L*_A,f,5_, *L*_A,f,max_, and *L*_PN,5_ at r ≥ 0.68. Roughness (*R*_m_ and *R*_5_), sharpness (*S*_5_) and fluctuation strength (*F*_5_) are weakly correlated (0.42 ≥|r| ≥ 0.3), where F_5_ is the only measurement that is negatively correlated with annoyance.

Figure 12 presents the correlation coefficients between the annoyance ratings of the noise from the flyby tests and the various noise metrics. Similar to the hover cases, annoyance is strongly correlated with *L*_A,f,max_ as well as *L*_EA_ and *L*_TPN,max_ at r ≥ 0.7, followed by *R*_5_, *N*_5_, and PA at r ≥ 0.63. *S*_5_ is weakly correlated (r=0.35), and there is little to no correlation for *F*_5_ (r=0.18).

From the correlation analysis, it can be seen that annoyance is relatively strongly correlated with the sound pressure level or loudness-related metrics, which in turn, were very strongly correlated with each other. However, the annoyance rating results from the previous section and the fact that no loudness-related metrics yielded a correlation coefficient above 0.71 suggest that the sound pressure level or loudness is not the sole explanation for how listeners rated the annoyance caused by the noise. There may possibly be other psychoacoustic-related metrics, such as roughness and sharpness, which although less correlated with both the other level metrics and annoyance, are needed to accurately model the annoyance caused by UAV noise events. However, Zwicker’s psychoacoustic annoyance (PA) metric did not correlate more strongly with the annoyance ratings than a sound pressure level based metric such as *L*_A,eq_. Di et al. [32] have shown that PA does not accurately estimate the annoyance caused by noise with prominent tonal components, and this may be the case for UAV noise.

### 5.4. Effect of L_A,f,max_ on Annoyance Rating

**Figure 13:**
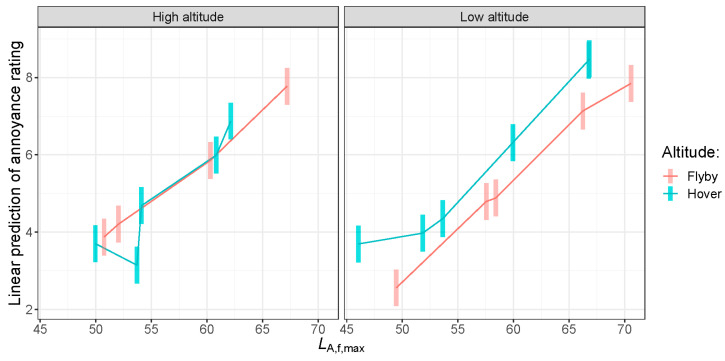
Linear prediction of the annoyance rating from the linear mixed model analysis using *L*_A,f,max_ as a predictor.

A model with a three-way interaction between the *L*_A,f,max_ metric, UAV altitude, and flying mode (hover and flyby) as fixed effects was used to examine the relationship between *L*_A,f,max_ and the annoyance rating. *L*_A,f,max_ was chosen from the list of objective metrics as it had a strong correlation with the annoyance ratings (see Figure 11 and Figure 12) and it could be measured for both flight modes. In addition, it is a well-known and easy-to-calculate metric that is currently used for the noise certification of propeller-driven light airplanes and light helicopters. Figure 13 plots the annoyance rating predicted using *L*_A,f,max_, for UAVs operating at different altitudes and flight modes. Each vertical error bar in Figure 13 shows the 95% confidence interval of annoyance rating for each *L*_A,f,max_ measurement. Each *L*_A,f,max_ measurement corresponds to one UAV at a particular flight condition and altitude. We observe that at a high altitude, apart from one UAV with *L*_A,f,max_ at 53 dB, there is no difference between flyby and hover in terms how *L*_A,f,max._ affects annoyance. That is, measuring *L*_A,f,max_ can predict a similar annoyance rating between flying modes. On the other hand, for low-altitude flight, the predicted annoyance produced by noise from a UAV in hover is higher than that produced by a UAV operating in flyby mode at a constant *L*_A,f,max_.

This analysis showed that *L*_A,f,max._ correlates closely with the annoyance rating, where, in general, the higher *L*_A,f,max._ is, the more annoying listeners perceive it to be. However, this is not to say that *L*_A,f,max._ is the only indicator of annoyance. There may be other factors that can affect annoyance, as shown by the increased annoyance produced by UAVs operating in hover compared to flyby at the same *L*_A,f,max_.

In Section 5.2, we noted that there was no evidence of significant differences between annoyance produced by the Mavic (*L*_A,f,max_ = 51.8 dB) and the Tello (*L*_A,f,max_ = 46.1 dB) hovering at low-altitude, despite the difference between their *L*_A,f,max_ values being 5.7 dB. This could be explained by the results shown in Figure 13 where we observe a plateauing of the annoyance rating at lower sound pressure levels for the hover noise events. This observation is in line with previous studies where the relationship between annoyance rating and loudness often follows a logistic law [60,61].

### 5.5. Cognitive Distraction Test Score Analysis

**Figure 14:**
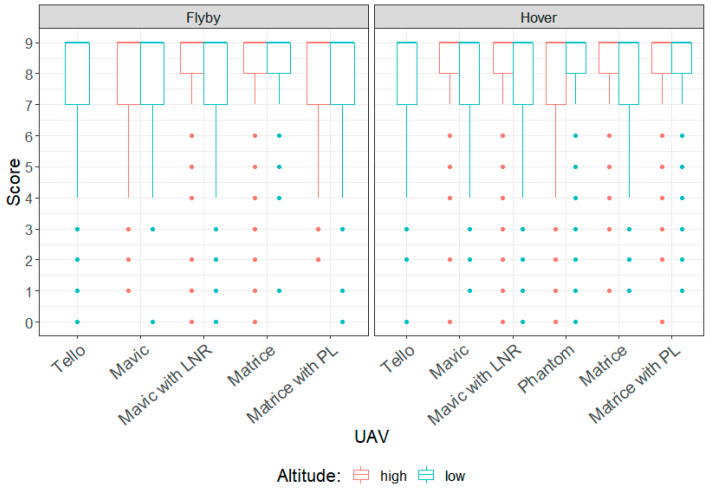
Scores from cognitive distraction test.

The scores for the cognitive distraction tests were calculated by counting the number of digits correctly recalled in the correct position within each sequence. The raw scores out of a total score of 9 for the tests conducted with noise from the different UAVs, flown in both hover or flyby modes, and at high altitude (30 m or 27 m) or low altitude (10 m), are presented as box plots in Figure 14. Note that as with the annoyance test, no data were collected for the Tello operating at high altitude, nor the DJI Phantom operating in flyby mode. Additionally, a high rating corresponds to a more accurate recall of the digits, and is thus considered to be an indication of the listener being less distracted. One participant did not enter in any answers, resulting in zero scores for all conditions. Their results were excluded from the analysis.

A model with a three-way interaction between the UAV types, altitude, and flying conditions (hover and flyby) as fixed effects was used to analyse the distraction scores. The participant ID was included as a random effect to account for the repeated measure in the test design. We found a significant three-way interaction using the model ($\chi^{2}\left(3\right)=16.7$, *p*-value < 0.001) from a likelihood ratio comparison by comparing the full model with a model without the interaction.

**Figure 15:**
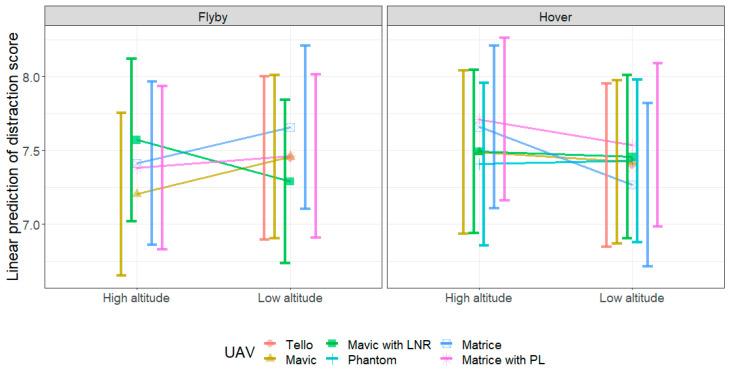
Linear prediction of distraction scores from the linear mixed model analysis in terms of altitude conditions. The error bars represent the 95% confidence interval of the linear prediction.

Figure 15 shows the predicted probabilities of distraction scores from the LME model. Table A3 presents the respective pairwise contrasts of the three-way interaction using *emmeans* within and between UAV types. From Table A3, we observe most contrasts within noise events produced by a particular UAV to be not significant—that is, the distraction scores in the current experiment were not significantly different between flight mode and altitude conditions. There were marginal significant differences for the distraction caused by the noise from the Mavic UAV between flyby and hover at high altitude, and for the Mavic with LNR between high and low altitudes for flyby, where in both cases the flyby at high altitude resulted in a lower performance from the participants. Participants performed significantly better during tests conducted using noise from the Matrice, both in flyby at a low altitude, and hovering at a high altitude compared to hover at low altitude. For the Matrice with PL at a high altitude, flyby was more distracting than hover. This is shown in Figure 15, where while an interaction is clear between the flight mode and altitude conditions, there does not appear to be much difference in the overlap of the confidence intervals within the same condition.

Between the different UAVs, there were only significant differences between the distraction caused by the noise from the Mavic and Mavic with LNR at the high-altitude flyby condition, where the tests using noise from the Mavic with LNR produced less distraction than those conducted with the noise from the Mavic (without LNR) by a score of 0.37 (*p* = 0.01). This is summarised in Table A4. However, the Mavic with LNR at the low-altitude flyby condition was more distracting than the Matrice by a score of 0.38 (*p* = 0.01). There was no significant difference between the distraction caused by the noise from any UAV in the hover condition at both high and low altitudes.

No objective metrics correlated significantly with the distraction scores for both the hover and flyby flight modes.

While the results showed that some UAVs or conditions can be more distracting, we found that in general the participants performed well regardless of the noise. This meant that the test results suffered from a ‘ceiling effect’, in which many of the test scores were at or close to the maximum and therefore differences in the effect caused by different noise events could be difficult to identify. We therefore conclude that the digit span test was not a suitable test to examine how UAVs at different flight and altitude conditions distract a listener. The results also did not correlate with any objective metrics. This is possibly a product of the test design, rather than distraction not being related to the objective metrics. However, the fact that the Matrice (*L*_A,f,max_ = 66.2 dB) was less distracting than the Mavic with LNR (*L*_A,f,max_ = 58.4 dB) for low-altitude flyby may suggest that distraction does not solely depend on the noise level, but may involve other acoustic features of the signal.

## 6. Conclusions and Recommendations

This paper presented a study investigating the perception of UAV noise for a range of different devices under different operating conditions, including flying mode and height. Measurements of the noise from the UAVs made using a ½″ microphone were used to calculate various noise metrics for each noise event. The noise was also recorded using a spherical microphone array. These recordings were reproduced using a HOA based 3D sound reproduction system in a large anechoic chamber at The University of Auckland. Participants in this study were subjected to the reproduced noise recordings and asked to rate their levels of annoyance in response to them and also to perform a simple cognitive test in order to assess distraction caused by the UAV noise.

The measurements made using the ½″ microphone were affected by ground reflections. Although such an effect would also occur for a human listening to UAV noise, it does mean that the measurements are dependent on the ground surface impedance. A certification test method for quantifying UAV noise should specify the ground impedance and flatness of the ground surrounding the test area. Alternatively, measurements could be made using a microphone mounted on a rigid sound board at ground height, as is common for other environmental noise measurement methods.

The noise level produced by the UAVs appears to be highly directional and therefore a UAV noise certification test should measure the noise produced by a UAV at multiple different locations.

The noise level produced by a UAV is also strongly dependent on the weight of the UAV, with heavier UAVs producing significantly higher noise levels than lighter UAVs. Our results appear to be consistent with the scaling law suggested in [45].

Statistical analysis of the results of the annoyance tests suggests that the effect of the flying mode and UAV height affect how the participants perceived the UAV noise. The relationship between the annoyance rating and the UAV type, altitude and flying mode is complex. However, generally, it was found that the low-altitude cases were more annoying than the corresponding high-altitude cases (for a given UAV and flying mode). This result is expected as for the low-altitude cases, the UAV was closer to the observation location and generally produced much higher levels of noise at this point. This finding was inconsistent with that of [5], who reported that there was no significant difference between the annoyance responses to noise produced by a UAV operating at different heights between 20 and 100 m above ground level, despite an 8 dB range in the *L*_EA_ for these cases.

In terms of the level of annoyance experienced from exposure to the noise from each UAV, the low-noise rotors did not seem to affect the annoyance rating of the noise produced by the Mavic UAV (there was no difference observed in any of the tests, except for the case of hover at high altitude where the UAV with low-noise rotors was found to be slightly more annoying). The Matrice was deemed significantly more annoying when a payload was added during both the hover and flyby conditions at high altitude. However, no significant difference was found in the average annoyance rating between the cases with or without the PL during hover at low altitude—and both of these cases produced the highest annoyance rating for the cases considered in this study. The Tello was found to be the least annoying of all of the UAVs for the low-altitude flyby. This result was not unexpected as this UAV was the smallest (and therefore produced the lowest levels of noise) amongst the UAVs tested. It is noteworthy that the annoyance produced by the Tello was not significantly different from the Mavic when hovering at low altitude.

The correlation analysis of the annoyance ratings and the objective metrics suggests that annoyance is strongly correlated with loudness-related metrics. However, both the correlation analysis and the annoyance rating analysis suggest that in order to model UAV noise annoyance, it may be necessary to look beyond loudness. For example, future work should investigate the effect of the spectral and temporal information of the noise signal on annoyance and should further investigate whether other qualities such as tonality and impulsiveness play a role in the annoyance caused by UAV noise.

The strong correlation between the annoyance ratings for the noise produced by the UAV in hover mode and in flyby mode suggests that only one form of flying condition (e.g., just hover or just flyby) may be needed for measurements to quantify the impact of noise from UAVs on humans (e.g., for certification testing). Analysis between *L*_A,f,max_ and annoyance showed that at a high altitude, the relationship between *L*_A,f,max_ and annoyance is not affected by the flight mode. However, at a low altitude, hovering is more annoying than flyby at the same *L*_A,f,max_ level.

The purpose of the cognitive distraction test was to measure how distracting UAV noise may be and to provide supporting evidence for the subjective annoyance ratings. Unfortunately, the results of this test were somewhat inconclusive and appear to have been affected by a ceiling effect—where participants scored consistently close to the maximum value in the test regardless of the noise. This made differences in distraction caused by different noise events difficult to identify. Nevertheless, some significant differences were observed. For the noise produced by the Mavic with LNR in flyby and the Matrice in hover, we observed that the noise produced at high attitudes was less distracting than that produced at low altitudes, corroborating the annoyance results. However, other differences were not consistent with the annoyance results. For example, the Matrice was less distracting than the Mavic with LNR in low-altitude flyby despite producing noise with a higher sound pressure level.

In addition, the results obtained from the experiment may not translate to real-world settings as the setup in the anechoic chamber would have been unfamiliar to most of the participants and the experiments did not include the visual aspect of the UAV encounter. Hence, the results observed in the current study may be specific to our test environment. We therefore plan to conduct future tests using a VR headset in order to improve the realism of the experiments (similar to the approach used in [6]). Additionally, further investigation is also required to confirm whether distraction from UAV noise may be caused by some other feature of the noise signal that is different to that leading to annoyance. Future work will involve devising a more suitable cognitive distraction task where we can accurately examine the differences in the distraction caused by UAV noise and also investigate if there is any relationship between this distraction and objective measures of the UAV noise.

## Figures and Tables

**Table 1:** Test details. Key: SLF = straight-and-level flight; LNR = low-noise rotors; PL = tested with a 0.461 kg payload.

No.	UAV	Flight Condition	Height of UAV	No.	UAV	Flight Condition	Height of UAV
1	Tello	SLF	10 m	11	Phantom	Hover	10 m
2	Tello	Hover	10 m	12	Phantom	Hover	27 m
3	Mavic	SLF	10 m	13	Matrice	SLF	10 m
4	Mavic	SLF	30 m	14	Matrice	SLF	30 m
5	Mavic	Hover	10 m	15	Matrice	Hover	10 m
6	Mavic	Hover	30 m	16	Matrice	Hover	30 m
7	Mavic with LNR	SLF	10 m	17	Matrice with PL	SLF	10 m
8	Mavic with LNR	SLF	30 m	18	Matrice with PL	SLF	30 m
9	Mavic with LNR	Hover	10 m	19	Matrice with PL	Hover	10 m
10	Mavic with LNR	Hover	30 m	20	Matrice with PL	Hover	30 m

## Appendix A

**Table A1:** Pairwise contrasts of the annoyance ratings within the UAV types. *p*-values in bold represent statistical significance (<0.05).

Tello					
Contrast	Estimate	SE	df	t-ratio	*p*-value
low flyby–low hover	−1.1351	0.162	2202	−7.017	**<0.0001**
Mavic					
Contrast	Estimate	SE	df	t-ratio	*p*-value
high flyby–low flyby	−0.9189	0.162	2202	−5.681	**<0.0001**
high hover–low hover	−0.8288	0.162	2202	−5.124	**<0.0001**
high flyby–high hover	0.7297	0.162	2202	4.511	**<0.0001**
low flyby–low hover	0.8198	0.162	2202	5.068	**<0.0001**
Mavic LNR					
Contrast	Estimate	SE	df	t-ratio	*p*-value
high flyby–low flyby	−0.6757	0.162	2202	−4.177	**0.0002**
high hover–low hover	−0.6486	0.162	2202	−4.01	**0.0004**
high flyby–high hover	0.5045	0.162	2202	3.119	**0.0099**
low flyby–low hover	0.5315	0.162	2202	3.286	**0.0057**
Phantom					
Contrast	Estimate	SE	df	t-ratio	*p*-value
high hover–low hover	−1.6306	0.162	2202	−10.08	**<0.0001**
Matrice					
Contrast	Estimate	SE	df	t-ratio	*p*-value
high flyby–low flyby	−1.2793	0.162	2202	−7.908	**<0.0001**
high hover–low hover	−2.4955	0.162	2202	−15.427	**<0.0001**
high flyby–high hover	−0.1351	0.162	2202	−0.835	0.8376
low flyby–low hover	−1.3514	0.162	2202	−8.354	**<0.0001**
Matrice PL					
Contrast	Estimate	SE	df	t-ratio	*p*-value
high flyby–low flyby	−0.0721	0.162	2202	−0.446	0.9705
high hover–low hover	−1.5766	0.162	2202	−9.746	**<0.0001**
high flyby–high hover	0.9009	0.162	2202	5.569	**<0.0001**
low flyby–low hover	−0.6036	0.162	2202	−3.731	**0.0011**

**Table A2:** Pairwise contrasts of the annoyance ratings between UAV types. *p*-values in bold represent statistical significance (<0.05).

High-Altitude Flyby					
Contrast	Estimate	SE	df	t-ratio	*p*-value
Mavic–Mavic with LNR	−0.3333	0.162	2202	−2.061	0.3086
Mavic–Matrice	−1.982	0.162	2202	−12.252	**<0.0001**
Mavic–Matrice with PL	−3.9009	0.162	2202	−24.115	**<0.0001**
Mavic with LNR–Matrice	−1.6486	0.162	2202	−10.192	**<0.0001**
Mavic with LNR–Matrice with PL	−3.5676	0.162	2202	−22.054	**<0.0001**
Matrice–Matrice with PL	−1.9189	0.162	2202	−11.862	**<0.0001**
Low-Altitude Flyby					
Contrast	Estimate	SE	df	t-ratio	*p*-value
Mavic–Mavic with LNR	−0.0901	0.162	2202	−0.557	0.9937
Mavic–Matrice	−2.3423	0.162	2202	−14.48	**<0.0001**
Mavic–Matrice with PL	−3.0541	0.162	2202	−18.88	**<0.0001**
Mavic–Tello	2.2342	0.162	2202	13.812	**<0.0001**
Mavic with LNR–Matrice	−2.2523	0.162	2202	−13.923	**<0.0001**
Mavic with LNR–Matrice with PL	−2.964	0.162	2202	−18.323	**<0.0001**
Mavic with LNR–Tello	2.3243	0.162	2202	14.369	**<0.0001**
Matrice–Matrice with PL	−0.7117	0.162	2202	−4.4	**0.0002**
Matrice–Tello	4.5766	0.162	2202	28.292	**<0.0001**
Matrice with PL–Tello	5.2883	0.162	2202	32.691	**<0.0001**
High-Altitude Hover					
Contrast	Estimate	SE	df	t-ratio	*p*-value
Mavic–Mavic with LNR	−0.5586	0.162	2202	−3.453	**0.0075**
Mavic–Phantom	−1.5405	0.162	2202	−9.523	**<0.0001**
Mavic–Matrice	−2.8468	0.162	2202	−17.599	**<0.0001**
Mavic–Matrice with PL	−3.7297	0.162	2202	−23.057	**<0.0001**
Mavic with LNR–Phantom	−0.982	0.162	2202	−6.07	**<0.0001**
Mavic with LNR–Matrice	−2.2883	0.162	2202	−14.146	**<0.0001**
Mavic with LNR–Matrice with PL	−3.1712	0.162	2202	−19.604	**<0.0001**
Phantom–Matrice	−1.3063	0.162	2202	−8.075	**<0.0001**
Phantom–Matrice with PL	−2.1892	0.162	2202	−13.533	**<0.0001**
Matrice–Matrice with PL	−0.8829	0.162	2202	−5.458	**<0.0001**
Low-Altitude Hover					
Contrast	Estimate	SE	df	t-ratio	*p*-value
Mavic–Mavic with LNR	−0.3784	0.162	2202	−2.339	0.1789
Mavic–Phantom	−2.3423	0.162	2202	−14.48	<0.0001
Mavic–Matrice	−4.5135	0.162	2202	−27.902	<0.0001
Mavic–Matrice with PL	−4.4775	0.162	2202	−27.679	<0.0001
Mavic–Tello	0.2793	0.162	2202	1.726	0.5143
Mavic with LNR–Phantom	−1.964	0.162	2202	−12.141	<0.0001
Mavic with LNR–Matrice	−4.1351	0.162	2202	−25.563	<0.0001
Mavic with LNR–Matrice with PL	−4.0991	0.162	2202	−25.34	<0.0001
Mavic with LNR–Tello	0.6577	0.162	2202	4.066	0.0007
Phantom–Matrice	−2.1712	0.162	2202	−13.422	<0.0001
Phantom–Matrice with PL	−2.1351	0.162	2202	−13.199	<0.0001
Phantom–Tello	2.6216	0.162	2202	16.206	<0.0001
Matrice–Matrice with PL	0.036	0.162	2202	0.223	0.9999
Matrice–Tello	4.7928	0.162	2202	29.628	<0.0001
Matrice with PL–Tello	4.7568	0.162	2202	29.405	<0.0001

**Table A3:** Pairwise contrasts of distraction scores for each UAV. *p*-values in bold represent statistical significance (<0.05).

	**Mavic** SE = 0.11, df = 7499			**Mavic with LNR** SE = 0.11, df = 7499−7500		
Contrast	Estimate	t-ratio	*p*-value	Estimate	t-ratio	*p*-value
High flyby–low flyby	−0.259	−2.31	0.0959	0.287	2.581	**0.0485**
Low flyby–low hover	0.037	0.329	0.9877	−0.172	−1.542	0.4124
High flyby–high hover	−0.289	−2.59	**0.0473**	0.078	0.702	0.8964
High hover–low hover	0.067	0.603	0.9312	0.038	0.34	0.9865
	**Matrice** SE = 0.11, df = 7499–7500			**Matrice with PL** SE = 0.11, df = 7499		
Contrast	Estimate	t-ratio	*p*-value	Estimate	t-ratio	*p*-value
High flyby–low flyby	−0.248	−2.218	0.1185	−0.081	−0.722	0.8885
Low flyby–low hover	0.397	3.563	**0.0021**	−0.077	−0.692	0.9001
High flyby–high hover	−0.250	−2.244	0.1116	−0.336	−3.009	**0.014**
High hover–low hover	0.399	3.598	**0.0018**	0.178	1.599	0.3792
	**Phantom** SE = 0.11, df = 7499			**Tello** SE = 0.11, df = 7499		
Contrast	Estimate	t-ratio	*p*-value	Estimate	t-ratio	*p*-value
Low flyby–low hover	n/a	n/a	n/a	0.052	0.463	0.9671
High hover–low hover	−0.023	−0.204	0.997	n/a	n/a	n/a

**Table A4:** Pairwise contrasts of distraction scores between the UAVs. *p*-values in bold represent statistical significance (<0.05).

	**Low, Hover**			**Low, Flyby**		
Contrast	Estimate	t-ratio	*p*-value	Estimate	t-ratio	*p*-value
Mavic–Mavic with LNR	−0.03521	−0.317	0.9996	0.17312	1.55	0.6317
Mavic–Phantom	−0.00755	−0.068	1	n/a	n/a	n/a
Mavic–Matrice	0.15791	1.422	0.7138	−0.20188	−1.808	0.4608
Mavic–Matrice with PL	−0.11671	−1.05	0.9008	−0.0027	−0.024	1
Mavic–Tello	0.02299	0.207	0.9999	0.00809	0.072	1
Mavic with LNR–Phantom	0.02766	0.25	0.9999	n/a	n/a	n/a
Mavic with LNR–Matrice	0.19312	1.74	0.5052	**−0.375**	**−3.37**	**0.0098**
Mavic with LNR–Matrice with PL	−0.0815	−0.734	0.9778	−0.17581	−1.574	0.6157
Mavic with LNR–Tello	0.0582	0.524	0.9952	−0.16503	−1.478	0.6787
Phantom–Matrice	0.16546	1.494	0.6684	n/a	n/a	n/a
Phantom–Matrice with PL	−0.10916	−0.985	0.9229	n/a	n/a	n/a
Phantom–Tello	0.03054	0.276	0.9998	n/a	n/a	n/a
Matrice–Matrice with PL	−0.27462	−2.473	0.1324	0.19919	1.783	0.4766
Matrice–Tello	−0.13492	−1.216	0.8294	0.20997	1.88	0.4146
MatricePL–Tello	0.1397	1.258	0.8079	0.01078	0.096	1
	**High, Hover**			**High, Flyby**		
Contrast	Estimate	t-ratio	*p*-value	Estimate	t-ratio	*p*-value
Mavic–Mavic with LNR	−0.0059	−0.053	1	**−0.37287**	**−3.339**	**0.0109**
Mavic–Phantom	0.08201	0.739	0.9771	n/a	n/a	n/a
Mavic–Matrice	−0.174	−1.57	0.6187	−0.21294	−1.901	0.4017
Mavic–Matrice with PL	−0.22747	−2.048	0.3152	−0.18059	−1.612	0.5906
Mavic with LNR–Phantom	0.08791	0.793	0.9688	n/a	n/a	n/a
Mavic with LNR–Matrice	−0.16811	−1.518	0.6523	0.15994	1.432	0.7075
Mavic with LNR–Matrice with PL	−0.22157	−1.998	0.3437	0.19228	1.722	0.5174
Phantom–Matrice	−0.25602	−2.31	0.1902	n/a	n/a	n/a
Phantom–Matrice with PL	−0.30948	−2.786	0.0597	n/a	n/a	n/a
Matrice–Matrice with PL	−0.05347	−0.482	0.9968	0.03234	0.289	0.9997
